# Musical Activity During Life Is Associated With Multi-Domain Cognitive and Brain Benefits in Older Adults

**DOI:** 10.3389/fpsyg.2022.945709

**Published:** 2022-08-25

**Authors:** Adriana Böttcher, Alexis Zarucha, Theresa Köbe, Malo Gaubert, Angela Höppner, Slawek Altenstein, Claudia Bartels, Katharina Buerger, Peter Dechent, Laura Dobisch, Michael Ewers, Klaus Fliessbach, Silka Dawn Freiesleben, Ingo Frommann, John Dylan Haynes, Daniel Janowitz, Ingo Kilimann, Luca Kleineidam, Christoph Laske, Franziska Maier, Coraline Metzger, Matthias H. J. Munk, Robert Perneczky, Oliver Peters, Josef Priller, Boris-Stephan Rauchmann, Nina Roy, Klaus Scheffler, Anja Schneider, Annika Spottke, Stefan J. Teipel, Jens Wiltfang, Steffen Wolfsgruber, Renat Yakupov, Emrah Düzel, Frank Jessen, Sandra Röske, Michael Wagner, Gerd Kempermann, Miranka Wirth

**Affiliations:** ^1^German Center for Neurodegenerative Diseases, Dresden, Germany; ^2^Section of Cognitive Neurophysiology, Department of Child and Adolescent Psychiatry, Faculty of Medicine, Technische Universität Dresden, Dresden, Germany; ^3^German Center for Neurodegenerative Diseases, Berlin, Germany; ^4^Department of Psychiatry, Charité – Universitätsmedizin Berlin, Berlin, Germany; ^5^Department of Psychiatry and Psychotherapy, University Medical Center, University of Göttingen, Göttingen, Germany; ^6^Institute for Stroke and Dementia Research, University Hospital, LMU Munich, Munich, Germany; ^7^German Center for Neurodegenerative Diseases, Munich, Germany; ^8^MR-Research in Neurology and Psychiatry, Georg-August-University Göttingen, Göttingen, Germany; ^9^German Center for Neurodegenerative Diseases, Magdeburg, Germany; ^10^Institute of Cognitive Neurology and Dementia Research, Otto-von-Guericke University, Magdeburg, Germany; ^11^German Center for Neurodegenerative Diseases, Bonn, Germany; ^12^Department for Neurodegenerative Diseases and Geriatric Psychiatry, University Hospital Bonn, Bonn, Germany; ^13^Bernstein Center for Computational Neuroscience, Charité – Universitätsmedizin, Berlin, Germany; ^14^German Center for Neurodegenerative Diseases, Rostock, Germany; ^15^Department of Psychosomatic Medicine, Rostock University Medical Center, Rostock, Germany; ^16^German Center for Neurodegenerative Diseases, Tübingen, Germany; ^17^Section for Dementia Research, Hertie Institute for Clinical Brain Research and Department of Psychiatry and Psychotherapy, University of Tübingen, Tübingen, Germany; ^18^Department of Psychiatry, Faculty of Medicine, University of Cologne, Cologne, Germany; ^19^Department of Psychiatry and Psychotherapy, Otto-von-Guericke University, Magdeburg, Germany; ^20^Systems Neurophysiology, Department of Biology, Darmstadt University of Technology, Darmstadt, Germany; ^21^Munich Cluster for Systems Neurology, Munich, Germany; ^22^Ageing Epidemiology Research Unit, School of Public Health, Imperial College London, London, United Kingdom; ^23^Department of Psychiatry and Psychotherapy, University Hospital, LMU Munich, Munich, Germany; ^24^Department of Psychiatry and Psychotherapy, Klinikum Rechts der Isar, Technical University Munich, Munich, Germany; ^25^Department for Biomedical Magnetic Resonance, University of Tübingen, Tübingen, Germany; ^26^Department of Neurology, University Hospital Bonn, Bonn, Germany; ^27^German Center for Neurodegenerative Diseases, Göttingen, Germany; ^28^Neurosciences and Signaling Group, Institute of Biomedicine, Department of Medical Sciences, University of Aveiro, Aveiro, Portugal; ^29^Excellence Cluster on Cellular Stress Responses in Aging-Associated Diseases, University of Cologne, Cologne, Germany; ^30^Center for Regenerative Therapies Dresden, Technische Universität Dresden, Dresden, Germany

**Keywords:** brain aging, resilience, cognitive reserve, prevention, brain plasticity, instrument playing

## Abstract

Regular musical activity as a complex multimodal lifestyle activity is proposed to be protective against age-related cognitive decline and Alzheimer’s disease. This cross-sectional study investigated the association and interplay between musical instrument playing during life, multi-domain cognitive abilities and brain morphology in older adults (OA) from the DZNE-Longitudinal Cognitive Impairment and Dementia Study (DELCODE) study. Participants reporting having played a musical instrument across three life periods (*n* = 70) were compared to controls without a history of musical instrument playing (*n* = 70), well-matched for reserve proxies of education, intelligence, socioeconomic status and physical activity. Participants with musical activity outperformed controls in global cognition, working memory, executive functions, language, and visuospatial abilities, with no effects seen for learning and memory. The musically active group had greater gray matter volume in the somatosensory area, but did not differ from controls in higher-order frontal, temporal, or hippocampal volumes. However, the association between gray matter volume in distributed frontal-to-temporal regions and cognitive abilities was enhanced in participants with musical activity compared to controls. We show that playing a musical instrument during life relates to better late-life cognitive abilities and greater brain capacities in OA. Musical activity may serve as a multimodal enrichment strategy that could help preserve cognitive and brain health in late life. Longitudinal and interventional studies are needed to support this notion.

## Introduction

Given that world populations are aging, age-related diseases such as Alzheimer’s disease (AD) are on the rise and pose public health challenges of utmost importance (Alzheimer’s Association, 2021). Healthy lifestyle activities are proposed to enhance brain and cognitive resources in older adults (OA) (Valenzuela et al., 2012; Wirth et al., 2014; Arenaza-Urquijo et al., 2015; Livingston et al., 2020), which may strengthen resilience against cognitive decline in aging and AD. Among others, regular musical activity, such as playing a musical instrument, is associated with reduced risk of developing dementia (Verghese et al., 2003; Balbag et al., 2014). To advance targeted intervention strategies, it is important to delineate presumed cognitive benefits and underlying brain correlates associated with musical activity in older age.

It has been proposed that musical activity shares communalities with the concept of environmental enrichment (Fauvel et al., 2014b; Sihvonen et al., 2017). In animal models, far-reaching neurobiological and behavioral benefits of environmental enrichment have been shown (Kempermann, 2019a). Playing a musical instrument is a highly stimulating activity that inherently combines complex stimulations involving the simultaneous perception and integration of multimodal motor, sensory, cognitive, emotional, and social stimulations (Lappe et al., 2008). Active engagement in this multimodal leisure activity is thus proposed to facilitate beneficial brain and cognitive plasticity throughout life until old age (Wan and Schlaug, 2010).

Indeed, there is evidence to suggest that musical activity may preserve or even enhance cognitive abilities that decline in older age (Román-Caballero et al., 2018; Sutcliffe et al., 2020). For example, it has been shown that OA with musical activity (both professionals and/or amateurs) outperform controls in multiple (far-transfer) cognitive domains including executive functions, attention, language, processing speed, visuospatial and/or memory abilities (Hanna-Pladdy and MacKay, 2011; Parbery-Clark et al., 2011; Hanna-Pladdy and Gajewski, 2012; Gooding et al., 2014; Mansens et al., 2018; Strong and Mast, 2019; Gray and Gow, 2020). Moreover, some of these cognitive benefits, in particular processing speed, appear to be particularly pronounced in older participants playing a musical instrument as compared to singing (Mansens et al., 2018). While most of the existing studies are cross-sectional in nature, presumed cognitive benefits of musical activity in older age are supported by musical intervention/training studies. According to such studies in musically non-experienced OA, learning how to play a musical instrument has a robust positive impact on higher-order cognitive abilities including executive functions, working memory, speech perception and visual memory compared to control conditions (Bugos et al., 2007; Seinfeld et al., 2013; Degé and Kerkovius, 2018; Worschech et al., 2021).

Comparatively little is known about the neural underpinnings of musical activity in OA (Schneider et al., 2019). Both, cross-sectional and longitudinal studies have mainly investigated respective brain correlates in young and middle-aged participants with musical activity (both professionals and amateurs) compared to controls. In this population, musical activity seems to be associated with brain plasticity, as e.g., reflected by greater gray matter volume (GMV) as well as white matter (WM) integrity in dedicated brain regions (Wan and Schlaug, 2010; Herholz and Zatorre, 2012). Among others, these areas comprise frontal and temporal GM and WM structures (Sluming et al., 2002; Gaser and Schlaug, 2003; Halwani et al., 2011; Gärtner et al., 2013; Fauvel et al., 2014a; Groussard et al., 2014; James et al., 2014), which are also strongly affected by healthy and pathological aging (Jack et al., 1997; Habes et al., 2016; Wirth et al., 2018). Musical activity is further positively associated with hippocampal structure and function in younger musicians (professionals and/or amateurs) based on cross-sectional (Groussard et al., 2010, 2014; Oechslin et al., 2013) and intervention/training (Herdener et al., 2010) studies.

In the same line, sparse investigations are suggestive of beneficial brain–behavior relationships relating greater GM and WM integrity to musical activity in OA. Higher levels of musical activity are correlated with larger GMV in frontal (inferior) and temporal (parahippocampal) brain regions in OA with various musical experiences (Chaddock-Heyman et al., 2021). Moreover, larger GMV in frontal (inferior) and temporal (hippocampal and superior temporal) regions has been found in a lifespan sample of professional piano tuners compared to controls (Teki et al., 2012). Another study showed greater WM microstructural integrity in the superior longitudinal fasciculus and uncinate fasciculus in aging musicians (Andrews et al., 2021). Finally, recent intervention/training studies in musically non-experienced OA have reported enhanced neural efficiency in a distributed frontal, temporal, and parietal network (Guo et al., 2021), stabilized WM microstructural integrity in the fornix (Jünemann et al., 2022) and increased cortical thickness in auditory brain regions (Worschech et al., 2022) in response to musical instrument playing compared to control conditions.

The main goal of the present cross-sectional study was to shed light on the association between participating in musical activity and indicators of brain and cognitive health in OA. Existing findings propose that regular musical activity, in particular playing a musical instrument (Mansens et al., 2018), may have benefits for late-life cognitive abilities. There is further indication that musical activity is associated with neurophysiological correlates of cognitive reserve and/or brain reserve in older age (Sutcliffe et al., 2020). Together, these mechanisms could contribute to better cognitive abilities (Chaddock-Heyman et al., 2021) and help counteract brain pathology (Stern et al., 2020) in late life, warranting further investigations. Here, we combined measures of long-term musical activity (as assessed by the self-reported frequency of playing a musical instrument across three life periods), multi-domain cognitive abilities and regional brain morphology to investigate the association and interplay among these variables in cognitively unimpaired OA. We hypothesized that musical activity is associated with better late-life abilities in multiple cognitive domains as well as larger GMV in pre-selected frontal, temporal and hippocampal regions and at the voxel level. We further anticipated that musical activity could be associated with a more efficient use of structural brain capacities, which may convey resilience in late life.

## Materials and Methods

### Overall Study Approach

To address our research questions, the present study assembled data from the ongoing, multi-center, observational DZNE-Longitudinal Cognitive Impairment and Dementia (DELCODE) study (Jessen et al., 2018) using a unique methodological approach: (1) Musical activity was assessed in a large sample of cognitively unimpaired OA (aged ≥ 60 years) as a binary group variable (i.e., participants with musical activity and well-matched controls) based on the self-reported frequency of playing a musical instrument across three life periods (i.e., young adulthood, mid-life, and late-life). (2) Cognitive abilities were assessed using sensitive latent factor composite scores across five cognitive domains and a global cognitive score (Wolfsgruber et al., 2020). (3) Brain morphology was assessed within pre-selected regions-of-interest (ROI) and at the voxel level to identify the spatial pattern of GMV associated with musical activity. (4) We accounted for reserve proxies (Stern, 2009) of high educational attainment, crystallized intelligence, socioeconomic status (SES) and participation in physical activity, most of which were considered in earlier studies on musical activity and cognitive abilities (Hanna-Pladdy and MacKay, 2011; Gooding et al., 2014; Mansens et al., 2018; Gray and Gow, 2020). In addition, we controlled for these potential confounders in respective associations with brain morphology.

### Participants

The sample used in the present study was obtained from the baseline DELCODE study. The overall design of the DELCODE study is explained in the supplement. In the present study, cognitively unimpaired participants, i.e., OA, first-degree relatives of AD patients (family history, FH), and participants with subjective cognitive decline (SCD), were included and merged across the three groups to increase the final sample size and statistical power. Recruitment procedures and inclusion criteria are described in detail elsewhere (Jessen et al., 2018). In brief, all participants were aged ≥ 60 years, German speaking, and provided informed consent. Normal cognitive function was defined as a test performance within –1.5 standard deviations of age-, sex- and education-adjusted norms on all subtests of the Consortium to Establish a Registry of Alzheimer’s Disease (CERAD) test battery (Morris et al., 1989). Exclusion criteria for OA, FH, and SCD were comprised of medical conditions including current or past major medical, neurological, or psychiatric disorders.

The DELCODE baseline dataset (total: *n* = 1079, data release for this study: 01.2021) was used to select a subset of participants into the present study. The selection procedure is detailed in the supplement (Supplementary Figure 1). We were able to include *n* = 70 older participants that reported musical activity across the life course (group of interest) and a well-matched control group (*n* = 70, for methodological details see below).

### Measurements

#### Measurement of Musical Activity

We operationalized musical activity by the self-reported frequency of playing a musical instrument across three life periods ranging from young adulthood (13 30 years), mid-life (30–65 years) to late-life (65 years onwards). The approach was chosen, because participation in musical activity across the life course might be particularly beneficial with brain plasticity thought to continue throughout life (Wan and Schlaug, 2010). In agreement with a previous large-scale population study (Mansens et al., 2018), we used the available self-reported information to assess participation in musical activity. In the DELCODE cohort, musical activity was measured with the Lifetime of Experiences Questionnaire (LEQ, Valenzuela and Sachdev, 2007) adapted for the German population (Roeske et al., 2018). Details on the quantification and analysis of musical activity are provided in the supplement.

In brief, for each life period, the frequency of musical activity was measured using the respective LEQ item (“How often did you play a musical instrument?”) with responses provided on a 6-point Likert scale (0/‘never,’ 1/‘less than 1 time per month,’ 2/‘1 time per month,’ 3/‘2 times per month,’ 4/‘weekly,’ 5/‘daily’). We constructed a coding scheme to assess musical activity during life as a binary variable that was comprised of two groups, similar to the previous study (Mansens et al., 2018): (1) The musical activity group (group of interest) included participants that reported having played a musical instrument during life. All participants started in early adulthood and continued playing until the current life period with higher frequency (‘2 times per month’ or more) in at least one life period, given their respective age. Notably, a total of *n* = 67 started playing a musical instrument with higher frequency in young adulthood, *n* = 47 played a musical instrument with higher frequency in at least two life periods and *n* = 23 played a musical instrument with higher frequency in one life period. A detailed graphical description showing the individual trajectories of musical activity across the given life periods is provided in the supplement (Supplementary Figure 2). (2) The control group included participants that reported to never have played a musical instrument in any of the given life periods.

#### Measurement of Cognitive Abilities

Cognitive abilities were assessed using latent composite scores of five cognitive domains, namely (1) learning and memory, (2) working memory, (3) executive functions and mental processing speed, (4) language, and (5) visuospatial abilities and a global cognitive score (i.e., the mean across the five cognitive domains). The composite scores were created by using a confirmatory factor analysis on neurocognitive tests from the extensive neuropsychological test battery in the DELCODE cohort, as described (Wolfsgruber et al., 2020) and applied (e.g., Amaefule et al., 2021; Ballarini et al., 2021; Wesselman et al., 2021) in previous reports. Information on the methodological procedure are provided in the supplement. Notably, the construction of latent factors scores across multiple neuropsychological tests is an established procedure (Hedden et al., 2012; Benson et al., 2018) with several advantages (Gross et al., 2015): Each latent factor represents a construct measured by the shared variance across multiple indicator variables. Thus, the latent variables are adjusted for measurement error and specificities of the individual tests. The method allows for an objective evaluation of cognitive performance across multiple tests and results can be generalized above the specific measurement methods.

The following neuropsychological tests contributed to each cognitive domain: (1) Learning and memory: Alzheimer’s Disease Assessment Scale-Cognitive Subscale (ADAS-cog) word list: trial 1, 2, 3, delayed recall and recognition, Free and Cued Selective Reminding Test (FCSRT) free recall and cue efficiency, Wechsler Memory Scale (WMS) logical memory 1 and 2, Consortium to Establish a Registry for Alzheimer’s Disease (CERAD) figure savings, Symbol-Digit-Modalities Test (SDMT) incidental learning, Face Name Test. (2) Working memory: Digit Span Forward and Backward, FCSRT interference task (Serial 3 s). (3) Executive functions: Trail Making Test (TMT) A and B, Number Cancelation, SDMT, Flanker Task. (4) Language: verbal fluency groceries and animals, Boston Naming Test (20 items), FCSRT naming. (5) Visuospatial abilities: Clock copying and drawing, CERAD Figure copying. Each cognitive composite score was z-transformed using the here-selected DELCODE baseline sample.

#### MRI Acquisition and Processing

The MRI data were acquired using 3-Tesla MRI scanners (Siemens, Erlangen, Germany), including three TIM Trio systems, four Verio systems, one Skyra system, and one Prisma system. The extensive MRI protocol of the DELCODE study is described elsewhere (Jessen et al., 2018). For the present analysis, we used T1-weighted images (i.e., 3D GRAPPA PAT 2, 1 mm^3^ isotropic, 256 × 256 px, 192 slices, sagittal, ∼ 5 min, TR 2500 ms, TE 4.33 ms, TI 110 ms, FA 7°) and T2-weighted images (i.e., 0.5 × 0.5 × 1.5 mm^3^, 384 × 384 px, 64 slices, orthogonal to hippocampal long axis, ∼12 min, TR 3500 ms, TE 353 ms, optimized for volumetric assessment of the medial temporal lobe). All scans underwent quality assessment provided by the DZNE imaging network (iNET, Magdeburg).

Regional GMV analysis was conducted in pre-selected ROI robustly affected by healthy and pathological aging due to AD. Based on prior findings (Jack et al., 1997; Habes et al., 2016; Wirth et al., 2018), we chose three ROIs comprising the frontal lobe, the temporal lobe and the hippocampus. Cortical GMV was evaluated as a global measure of brain integrity. For each of the ROIs, we used regional GMV measures provided in the DELCODE database, as described previously (Düzel et al., 2018). In brief, structural MRI images were segmented in native space using an automated cortical parcellation pipeline (Fischl et al., 2004) implemented in FreeSurfer^1^ (version 6.0) and an advanced segmentation tool (Iglesias et al., 2015) to derive ROI-based GMV measures. Frontal and temporal GMV were calculated as the sum over selected ROIs of the left and right hemisphere, as proposed by Desikan et al. (2006). Left and right hippocampal volume were summed as a measure of the overall hippocampal volume. Details on the ROI computation are provided in the supplement. Regional GMV measures were adjusted for total intracranial volume (TIV), as estimated using FreeSurfer (Buckner et al., 2004).

We further assessed GMV at the voxel level using the following procedure. Structural MRI images were segmented to extract gray matter (GM), white matter (WM), and cerebrospinal fluid (CSF) tissues using the unified segmentation algorithm in CAT12^2^ (version 12.6) with default parameters. Warping to the Montreal Neurological Institute (MNI) template space was performed using Diffeomorphic Anatomical Registration Through Exponentiated Lie Algebra (DARTEL) with default parameters and registration to existing templates (Ashburner, 2007). TIV was computed as the sum of volumes of GM, WM, and CSF using the SPM “Estimate TIV and global tissue volumes” routine. Voxel-based statistical analyses were performed on the warped and modulated GMV maps, which were smoothed by a three-dimensional Gaussian kernel with full width at half maximum of 8 mm^3^.

#### Additional Measures

Age, sex, education, diagnostic category and known reserve proxies of crystallized intelligence, SES, and self-reported participation in physical activity were considered as potential confounders. Educational attainment was measured in years of education. Crystallized intelligence was estimated using the Multiple-Choice Vocabulary Intelligence Test (MWT, min. score: 0, max. score: 37), with higher scores being proportionally related to a higher level of verbal intelligence (Lehrl, 2005). The MWT is considered an established tool for the assessment of crystallized intelligence. The SES was estimated for each participant using the international socio-economic index of occupational information score (ISEI, min. score: 16, max. score: 90) (Ganzeboom et al., 1992), based on the occupational history assessed by the LEQ. Details on the SES computation are provided in the supplement.

Participation in physical activity (long-term and current) was assessed using the respective items of the LEQ and the Physical Activity Scale for the Elderly (PASE, min. score: 0) (Washburn et al., 1999), respectively. Self-reported participation in long-term physical activity was measured based on LEQ responses on the frequency of physical activity recorded for each life period using the 6-point Likert scale (see above). A mean score was calculated over the available participant’s responses including at least two life periods. In addition, current physical activity was assessed through the Physical Activity Scale for the Elderly (PASE, min. score: 0) (Washburn et al., 1999). The PASE includes leisure, household and occupational activities measured over the previous week. Based on frequency, duration, and intensity of these activities, a total score is calculated with higher scores indicating greater levels of physical activity. Long-term physical activity was significantly correlated with current physical activity in the matched sample (*n* = 140, *r* = 0.35, *p* < 0.001), supporting the validity of the measure. Long-term physical activity was used as a covariate in statistical analyses, since the measure was available from all participants.

### Statistical Analyses

Statistical analyses were conducted using *R* (version 3.5.1.) and Statistical Parametric Mapping (SPM, version 12, Wellcome Trust Centre for Neuroimaging, London, United Kingdom). Figures were generated using the package *ggplot2* (Wickham, 2016). Before conducting statistical models, statistical assumptions were assessed visually using diagnostic plots.

#### Sample Characteristics

Participants reporting musical activity during life and controls with no musical activity were matched using a one-to-one matching procedure taking into account age, sex, diagnostic category, education, SES, crystallized intelligence, and long-term physical activity. Further details are provided in the supplement including sample descriptive of the pre-matching sample (Supplementary Table 1). The one-to-one matching procedure was carried out using propensity score matching with the *R* package *MatchIt* (version 4.1.0.) (Ho et al., 2011). This statistical matching technique aims to estimate treatment or intervention effects by accounting for several covariates. Observations were matched based on the nearest-neighbor method, as a simple and effective procedure for selecting well-matched groups (Stuart, 2010). After matching, musical activity groups were compared in baseline demographic, behavioral, neuropsychological, and neuroimaging variables. Independent Student’s *t*-tests were used for all continuous variable and chi-squared (χ^2^) tests were applied for all categorical variables.

#### Regions-of-Interest-Based Analyses

Multiple linear regression models were used to assess our main hypotheses. In these statistical analyses, an alpha value of 0.05 was considered statistically significant. In addition, correction for multiple comparisons was performed using a false discovery rate (FDR)-adjusted *p*-value threshold (alpha) of 0.05 (Benjamini and Hochberg, 1995). Uncorrected *p*-values were reported, when results survived FDR correction, this is highlighted in respective result tables.

Firstly, the association between the musical activity groups (modeled as a main effect) and cognitive abilities were assessed using the cognitive composite scores. Multiple linear regression models were computed including musical activity (binary group) as an independent variable and each cognitive composite score (*z*-transformed) as a dependent variable, respectively. Next, the association between musical activity and brain morphology was examined using similar multiple linear regressions. The models included musical activity (binary group) as independent variable and each ROI-based GMV (frontal, temporal and hippocampus, all TIV adjusted) as dependent variable along with scanner site as covariate (dummy coded). Selected associations were visualized to facilitate the interpretation of findings using box plots of unadjusted data.

Next, we assessed the moderation of musical activity on the association between ROI-based GMV and cognitive abilities. To do this, musical activity (binary group), ROI-based GMV (frontal, temporal, and hippocampal, all mean-centered), and the interaction (musical activity × ROI-based GMV) along with scanner site as covariate (dummy coded) were entered into multiple regression models with each cognitive composite score (*z*-transformed) as dependent variable. To specify the directionality of significant interactions, simple slope analyses were carried out (Aiken and West, 1991; Cohen et al., 2003). Moderation effects were visualized using unadjusted data as follows: cognitive composite scores (*z*-transformed) were graphed as a function of musical activity and ROI-based GMV, respectively.

#### Voxel-Based Analysis

To elucidate the spatial distribution of the associations between musical activity and brain morphology at the voxel level, multiple linear regressions were computed in SPM12. For the present purpose, voxel-wise results were presented at *p* < 0.001 uncorrected and, if applicable, *p* < 0.05 with family-wise error (FWE) correction at peak level, in combination with the estimated expected voxels per cluster (*k*), as automatically calculated by SPM.

First, a multiple regression model was computed with musical activity (binary group) as independent variable and the modulated, warped, and smoothed GMV maps as dependent variable. Next, a moderation of musical activity was evaluated at the voxel level. This multiple regression model included musical activity (binary group), the respective cognitive composite score (*z*-transformed), and the interaction term (musical activity × cognitive composite score) as independent variables with GMV maps as dependent variable. The later analysis was carried out for global cognition and all cognitive domains. For reasons of simplicity, results of this analysis were displayed for one cognitive domain (i.e., with the largest effect size), as selected by the strongest moderation effect in the ROI-based analysis. Additional findings were provided in the extended data documentation.

All voxel-based analyses were adjusted for TIV as well as scanner site (dummy coded) and restricted to cerebral GM using an explicit binary GM mask derived from the present sample (i.e., average GM map thresholded at a level of *>* 0.3, excluding cerebellum and brain stem). Cluster peaks are specified by anatomical site, as labeled using the Hammersmith atlas (Hammers et al., 2003) provided by the CAT12 toolbox. Additionally, Brodmann areas (BA) were identified for cluster peaks using the BioImage Suite Web 1.2.0^3^ (GitHub, Retrieved December 2, 2021). Finally, mean values were extracted for each participant within combined clusters using the warped, modulated, and non-smoothed GMV images using the MarsBaR toolbox (release: 0.44^4^) (Brett et al., 2002), to provide complementary visualizations of the associations.

## Results

### Sample Characteristics

This study included a total sample of 140 older participants (aged ≥ 60 years) selected from the DELCODE cohort. The present sample comprised 70 participants reporting participation in musical activity during life and 70 well-matched controls without musical activity. The two groups (musical activity, no musical activity) were comparable in age, sex, distribution of diagnostic categories as well as reserve proxies including higher educational attainment, crystallized intelligence, SES, and participation in both long-term and current physical activity (all *p’s* > 0.05, Table 1). Slight group differences in frontal and total GMV (unadjusted raw values) were observed, with larger volumes in the older participants with musical activity compared to controls. No significant differences were observed in the temporal and hippocampal GMV (unadjusted raw values).

**TABLE 1:** Descriptive characteristics of the matched DELCODE sample (*n* = 140).

	Musical activity	No musical activity	*P*-value
Number (*n*)	70	70	–
Age (years)	68.23 (6.62)	69.01 (5.44)	0.445
Gender female/male (*n*)	31/39	35/35	0.498
Education (years)	16.20 (2.71)	15.96 (2.74)	0.598
Diagnostic category OA/FH/SCD (n)	19/7/44	24/6/40	0.654
SES^a^	66.27 (16.32)	65.21 (16.04)	0.699
Crystalized intelligence^b^	33.31 (2.14)	33.04 (2.22)	0.463
Physical activity, long-term^c^	4.25 (0.78)	4.32 (0.71)	0.611
Physical activity, current^d^	33.86 (11.80), *n* = 66	32.45 (12.85), *n* = 69	0.507
Total frontal GMV (ml)	138.86 (12.44)	134.69 (11.88)	0.044*
Total temporal GMV (ml)	95.53 (8.44)	93.29 (8.3)	0.115
Total hippocampal GMV (ml)	6.26 (0.71)	6.21 (0.66)	0.692
Total cortical GMV (ml)	453.83 (37.56)	441.69 (37.13)	0.049*

Descriptive data are given if applicable as mean and standard deviation (in parenthesis). The actual sample size is provided, if different from sample size specified in first row. P-values correspond to independent t-tests for unequal variance with participant group as independent variable. Chi-square statistic was used to compare the distribution of categorical variables. *p < 0.05. OA, older adults; FH, participants with a family history of AD; GMV, gray matter volume; SCD, participants with subjective cognitive decline; SES, socioeconomic status.

*^a^International socio-economic index (ISEI); ^b^Multiple-Choice Vocabulary Intelligence Test (MWT); ^c^Lifetime of Experiences Questionnaire (LEQ); ^d^Physical Activity Scale for the Elderly (PASE).*

*PASE: The total score was calculated based on frequency, duration, and intensity of leisure, household and occupational activities. Higher scores correspond to greater levels of physical activity.*

*LEQ: The mean frequency of physical activity over available life periods was measured using a 6-point Likert scale (0 = ‘never,’ 1 = ‘less than 1 time per month,’ 2 = ‘1 time per month,’ 3 = ‘2 times per month,’ 4 = ‘weekly,’ 5 = ‘daily’). Higher scores correspond to greater frequencies of physical activity.*

### Musical Activity and Cognitive Abilities

**FIGURE 1:**
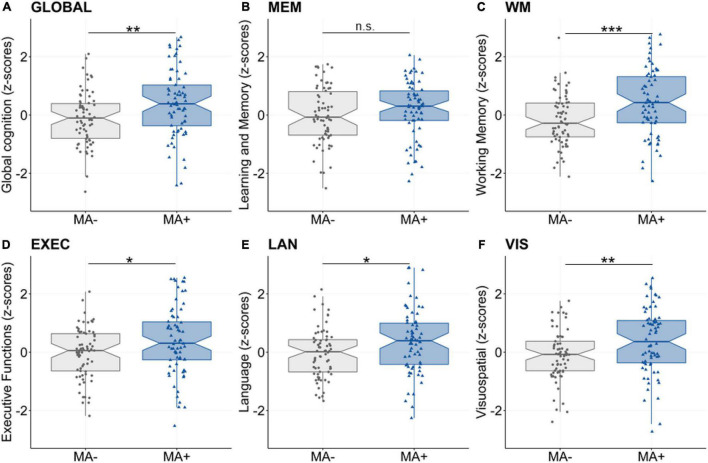
Main effect of musical activity on multi-domain cognitive abilities. Significant group differences were found for global cognition (**A**, GLOBAL), working memory (**C**, WM), executive function (**D**, EXEC), language (**E**, LAN), and visuospatial abilities (**F**, VIS). These multi-domain cognitive abilities were enhanced for participants with musical activity (MA+, blue) compared to controls (no musical activity across lifespan, MA-, gray). The association was not significant for the learning and memory composite (**B**, MEM). Boxplots display unadjusted data with individual data points. The “notch” shows the median with 95% confidence intervals and interquartile range with lower (25th) and upper percentiles (75th). Significance levels (uncorrected): ***p < 0.001, **p < 0.01, *p < 0.05. MA+, musical activity; MA–, no musical activity.

First, we assessed the association between musical activity and cognitive abilities using the latent composite scores. We found significant group differences for global cognition, working memory, executive function, language and visuospatial abilities (all *p’s* < 0.05, Table 2 and Figure 1). Performance in these cognitive abilities was significantly better in participants with musical activity compared to controls. In contrast, the two groups did not differ significantly the domain of learning and memory (*p* = 0.209).

**TABLE 2:** Results of linear regression analyses between musical activity and cognition.

	Dependent variable	Independent variable	*B*	SE B	Beta	*P*-value	Total *R*^2^ (adj.)
1	Global cognition	Musical Activity	0.540	0.178	0.250	0.003**^†^	0.062 (0.056)
2	Learning and Memory	Musical Activity	0.209	0.165	0.107	0.209	0.011 (0.004)
3	Working Memory	Musical Activity	0.669	0.178	0.304	< 0.001***^†^	0.092 (0.086)
4	Executive Functions	Musical Activity	0.465	0.180	0.214	0.011*^†^	0.046 (0.039)
5	Language	Musical Activity	0.443	0.170	0.216	0.010*^†^	0.047 (0.040)
6	Visuospatial	Musical Activity	0.522	0.176	0.245	0.003**^†^	0.060 (0.053)


Musical activity was included as binary predictor, dummy coded with musical activity = 1, no musical activity = 0. ***p < 0.001, **p < 0.01, *p < 0.05. ^†^p < 0.05 false discovery rate (FDR)-adjusted for statistical tests performed across cognitive domains. B, unstandardized coefficient; SE, standard error; Beta, standardized coefficient; R^2^, explained variance.

### Musical Activity and Brain Morphology in Regions-of-Interest

Second, we assessed the association between musical activity and regional GMV in pre-selected frontal, temporal and hippocampal ROIs (TIV-adjusted values). There were no significant group differences between participants with musical activity compared to controls in frontal (*p* = 0.822), temporal (*p* = 0.711), and hippocampal (*p* = 0.551) GMVs. Furthermore, the two groups did not differ significantly in total cortical GMV (*p* = 0.722). Results are shown in Table 3. In a *post hoc* analysis, the association between musical activity and frontal, temporal and hippocampal ROIs was analyzed separately for left and right hemispheres. There were no significant differences in the ROI-based GMVs between participants with musical activity and controls (all *p’s* > 0.1, Supplementary Table 2).

**TABLE 3:** Results of linear regression analyses between musical activity and GMV in regions-of-interest.

	Dependent variable	Independent variable	*B*	*SE* B	Beta	*P*-value	Total *R*^2^ (adj.)
1	Frontal GMV	Musical Activity	–0.046	0.204	–0.020	0.822	0.120 (0.052)
2	Temporal GMV	Musical Activity	–0.053	0.142	–0.032	0.711	0.123 (0.055)
3	Hpc GMV	Musical Activity	–0.007	0.012	–0.052	0.551	0.102 (0.032)
4	Cortical GMV	Musical Activity	–0.303	0.850	–0.031	0.722	0.132 (0.065)

*Models adjusted for scanner site. Musical activity was included as binary predictor, dummy coded with musical activity = 1, no musical activity = 0. Regional GMV was adjusted by total intracranial volume (TIV). B, unstandardized coefficient; Hpc, Hippocampus; SE, standard error; Beta, standardized coefficient; R^2^, explained variance; GMV, gray matter volume.*

### Moderations of Musical Activity in Regions-of-Interest

**FIGURE 2:**
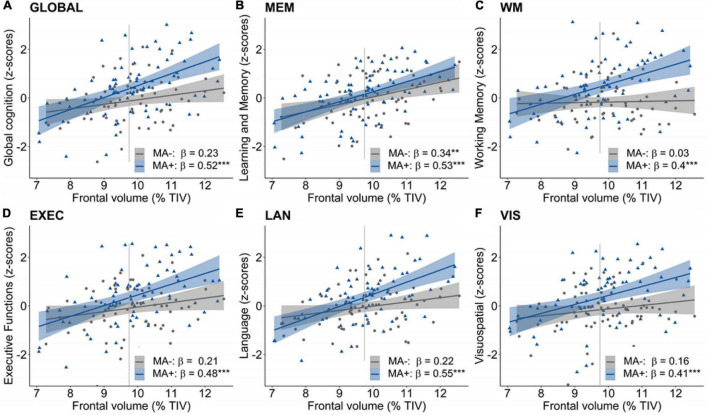
Moderation of musical activity in the frontal region. A significant moderation of musical activity was observed for global cognition (**A**, GLOBAL), working memory (**C**, WM), and language abilities (**E**, LAN), such that larger frontal GMV was associated with better global in participants with musical activity (MA+, blue) compared to controls (MA–, gray). This interaction was not significant for learning and memory (**B**, MEM), executive functions (**D**, EXEC), and visuospatial abilities (**F**, VIS). Individual data points (dots and triangles), linear trends (solid lines), 95% confidence intervals (shaded areas), and standardized regression coefficients (β) within each group are provided. Gray vertical lines display the 90th percentile of the prefrontal GMV distribution in AD patients of the DELCODE study. Significance levels (uncorrected): ***p < 0.001, **p < 0.01. GMV, gray matter volume; MA+, musical activity; MA–, no musical activity; TIV, total intracranial volume.

Third, a moderation of musical activity was assessed by the interaction between musical activity and GMV in the frontal, temporal and hippocampal ROIs, respectively. We found overall positive associations of frontal GMV with global and domain-specific cognitive abilities (all *p’s* ≤ 0.01, data not shown). Importantly, musical activity interacted with frontal GMV for global cognition, working memory, and language abilities (all *p’s* < 0.05; Table 4). Visualization of this moderation effect (Figure 2) showed that the association between frontal GMV and those cognitive abilities was enhanced in participants with musical activity compared to controls. That is, larger frontal GMV was significantly associated with better cognitive abilities in the participants with musical activity (all *p’s* < 0.05, Figure 2). The moderation effect was not detectable for the domain of learning and memory (*p* = 0.441).

**TABLE 4:** Results of the moderation analyses between musical activity and frontal GMV.

	Dependent variable	Independent variable	B	*SE* B	Beta	*P*-value	Total *R*^2^ (adj.)
1	Global cognition	Music Activity × frontal GMV	0.318	0.139	0.261	0.024*^†^	0.332 (0.269)
2	Learning and Memory	Music Activity × frontal GMV	0.102	0.132	0.092	0.441	0.263 (0.193)
3	Working Memory	Music Activity × frontal GMV	0.432	0.141	0.348	0.003**^†^	0.335 (0.273)
4	Executive Functions	Music Activity × frontal GMV	0.278	0.145	0.228	0.058	0.273 (0.204)
5	Language	Music Activity × frontal GMV	0.316	0.133	0.274	0.019*^†^	0.320 (0.256)
6	Visuospatial	Music Activity × frontal GMV	0.227	0.145	0.189	0.119	0.251 (0.180)

Models adjusted for scanner site. Musical activity was included as binary predictor, dummy coded with musical activity = 1, no musical activity = 0. Frontal GMV was adjusted for total intracranial volume and mean centered. ***p < 0.001, **p < 0.01, *p < 0.05. ^†^p < 0.05 false discovery rate (FDR)-adjusted for statistical tests performed across cognitive domains. B, unstandardized coefficient; SE, standard error; Beta, standardized coefficient; R^2^, explained variance.

For the temporal and hippocampal ROIs, we found overall positive associations of temporal GMV with global and domain-specific cognitive abilities (all *p*’s < 0.01, data not shown). For the temporal ROI, we detected a moderation of musical activity on the association of temporal GMV and global cognition, working memory and language abilities (all *p’s* < 0.05 uncorrected, Supplementary Table 3). There was no significant moderation of musical activity on the association between hippocampal GMV and cognitive abilities (all *p’s* > 0.1, Supplementary Table 4).

### Voxel-Based Analysis

**FIGURE 3:**
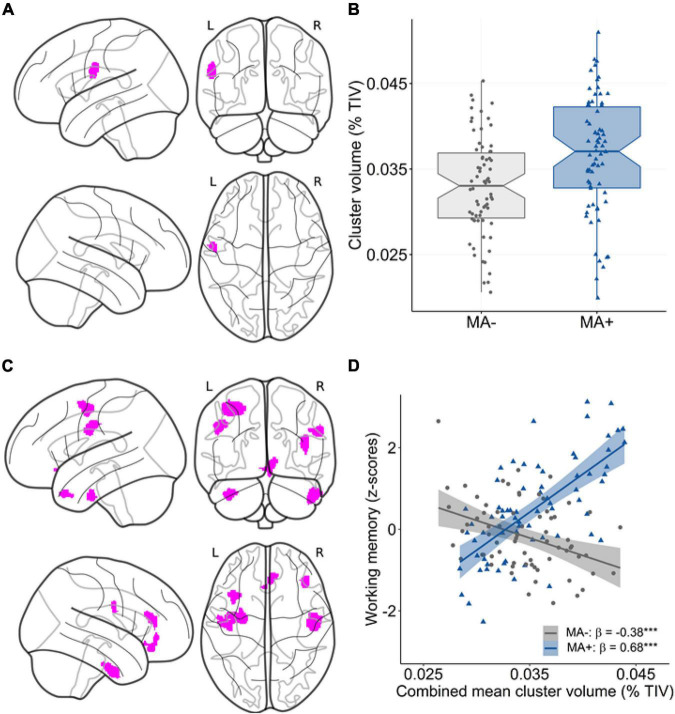
Association between musical activity and regional volume distribution. **(A,B)** Results of the main effect analysis. Statistical map **(A)** shows significant clusters (*p* < 0.001 uncorrected, color-coded in magenta) with larger GMV in participants with musical activity compared to controls. The corresponding graph **(B)** displays the association using mean GMV values extracted from the corresponding cluster in the postcentral gyrus. The box plot displays the median with 95% confidence intervals, interquartile range with lower (25th) and upper percentiles (75th), and individual data points. **(C,D)** Results of the moderation analysis. The statistical map **(C)** displays clusters (*p* < 0.001 uncorrected, color-coded in magenta) with a significant moderation effect of musical activity. The corresponding scatter plot **(D)** shows the association using mean values extracted from the GMV maps in the combined cluster. Larger GMV in the combined cluster was associated with better working memory ability selectively in participants with musical activity (MA+, blue) compared to controls (MA–, gray). Individual data points, linear trends (solid lines), 95% confidence intervals (shaded areas), and standardized regression coefficients (β) within each musical activity group are provided. The statistical maps are depicted on a glass brain. Significance levels (uncorrected): ***p < 0.001. MA+, musical activity; MA–, no musical activity; GMV, gray matter volume; TIV, total intracranial volume.

Our analysis at the voxel level largely confirmed the ROI-based findings. Regarding the main effect, there was a subtle positive association between musical activity and local GMV within a small cluster in the left postcentral gyrus (*p* < 0.001 uncorrected; Table 5 and Figures 3A,B). No other significant clusters were found. When investigating the moderation of musical activity at the voxel level, significant clusters of regional GMV were detected in prefrontal (lateral and medial), inferior temporal, and precentral regions (*p* < 0.001 uncorrected; Table 5). Visualization of this moderation effect for working memory (i.e., domain with the largest effect size) indicated that larger GMV was associated with better working memory ability only in participants with musical activity (Figures 3C,D). Overall, the results of the voxel-wise moderation analysis across all cognitive composites were essentially similar, with some variations in the number and location of significant clusters (Supplementary Table 5 and Supplementary Figure 3).

**TABLE 5:** Results of analyses between musical activity and GMV at the voxel level.

No. cluster	Label (Hammersmith atlas)	Brodmann area (BA)	Hemisphere	Cluster		Peak of cluster			
					
				*P*	size	*Z* value	MNI coordinates (*x y z*)		
**Main effect: musical activity^a^**									
1	Postcentral gyrus	BA 6	Left	0.195	220	3.60	–57	–6	27
**Interaction effect^b^**									
1	Precentral gyrus^†^	BA 6	Left	0.025	718	4.90	–28	–10	48
2	Precentral gyrus	BA 4	Left	0.114	322	4.81	–39	–14	33
3	Inferior middle temporal gyrus	BA 20	Right	0.054	502	4.45	51	–12	–42
4	Fusiform gyrus	BA 20	Left	0.279	145	3.99	–36	–8	–38
5	Inferior frontal gyrus	BA 46	Lateral/right	0.140	277	3.90	40	33	12
6	Anterior medial temporal lobe	BA 38	Left	0.245	168	3.78	–38	16	–36
7	Orbito-frontal lobe	BA 11	Medial/right	0.166	242	3.59	6	34	–14
8	Postcentral gyrus	BA 4	Right	0.293	137	3.52	50	–8	26

*Models adjusted for scanner site and TIV.*

*Musical activity was included as binary predictor, dummy coded with musical activity = 1, no musical activity = 0.*

*^a^Results from the main effect model with musical activity and GMV (p < 0.001 uncorrected, expected voxels per cluster k = 140).*

*^b^Results from the interaction effect model with musical activity, working memory, and GMV (p < 0.001 uncorrected, expected voxels per cluster k = 134).*

*^†^Significant after FWE correction (p < 0.05, expected voxels per cluster k = 42).*

*Cluster peaks are specified by their anatomical site, labeled using the Hammersmith atlas provided by the CAT12 toolbox.*

*Brodmann areas were identified with the BioImage Suite Web 1.2.0.GMV, gray matter volume; MNI coordinates (x y z), coordinates in MNI space in millimeters; TIV, total intracranial volume.*

## Discussion

### Summary of Findings

The current study examined cognitive abilities, brain morphology, and their interplay in OA that reported having played a musical instrument across three life periods. Participants with a history of long-term musical activity were compared to controls (i.e., without a history of musical instrument playing) that were closely matched for known reserve proxies. We document three main findings: (1) Participants with musical activity outperformed controls in global and multi-domain cognitive abilities, but not in learning and memory. (2) Participants with musical activity did not significantly differ from controls in GMV within the higher-order frontal, temporal, and hippocampal regions. (3) The association between GMV in distributed frontal-to-temporal regions and multi-domain cognitive abilities was enhanced in participants with musical activity compared to controls. Together, our correlational findings suggest that participation in musical activity during life is associated with brain and cognitive benefits in late life and could strengthen cognitive resilience. Longitudinal studies are needed to support this interpretation.

### Musical Activity and Cognitive Abilities

We show that musical activity during life is associated with better late-life cognitive abilities in OA. More precisely, participants with musical activity outperformed the matched controls in global cognition and multiple cognitive domains including working memory, executive functions, language and visuospatial abilities. These findings agree with a body of studies, suggesting that active participation in musical activity is associated with higher-order cognitive abilities in OA, based on correlational (Hanna-Pladdy and MacKay, 2011; Hanna-Pladdy and Gajewski, 2012; Mansens et al., 2018; Groussard et al., 2020) and intervention/training (Bugos et al., 2007; Seinfeld et al., 2013; Worschech et al., 2021) studies. A recent meta-analysis of active musical training further demonstrates a small but measurable benefit of this leisure-time activity on cognitive functioning in OA with mild cognitive impairment and dementia (Dorris et al., 2021).

In our study, the association between musical activity and late-life cognitive abilities was greatest for working memory (β = 0.304), a fundamental cognitive domain that is central to overall cognitive functioning (Baddeley, 2003). The result mirrors existing findings in younger (Talamini et al., 2017) and in older (Hanna-Pladdy and Gajewski, 2012; Grassi et al., 2017; Mansens et al., 2018) musically active adults and corroborates a particular involvement of frontal-lobe functions in playing music (Jäncke, 2013; Sutcliffe et al., 2020). In contrast, participants with musical activity did not differ from controls in the domain of learning and memory, although this must be perceived as an essential cognitive skill involved in playing a musical instrument (Talamini et al., 2017). Some correlational studies have reported that musical activity is indeed associated with better episodic memory in OA (Hanna-Pladdy and MacKay, 2011; Gooding et al., 2014; Mansens et al., 2018; Romeiser et al., 2021), while others do not find such effects (Hanna-Pladdy and Gajewski, 2012; Fauvel et al., 2014b; Strong and Mast, 2019). Taken together, our findings in OA substantiate that musical activity may predominantly favor cognitive abilities involving the frontal lobe.

One might argue that the present null result in learning and memory could be explained by a lack in sensitivity of our composite measure. However, this latent factor score was previously shown to capture even subtle inter-individual differences in memory performance of OA (Wolfsgruber et al., 2020). Alternatively, it is plausible that specific memory sub-processes are enhanced by musical/auditory expertise involving the tonal stimulus modality (Talamini et al., 2017), such as long-term musical memory (Groussard et al., 2010) or auditory navigation (Teki et al., 2012), which are not mapped by our memory composite score. Experimental, neuroimaging, and neuropsychological markers tapping into more specific hippocampal processes (e.g., Stark et al., 2019) will be needed to gain further insight into presumed memory benefits associated with musical activity in older age.

### Moderation of Musical Activity

As a novel finding, the present study documents a moderation of musical activity in OA. More specifically, we found that larger GMV was significantly associated with better multi-domain cognitive abilities in participants with musical activity compared to controls. This specific moderation was observed for global cognition, working memory, as well as language abilities mainly in the pre-selected frontal ROI and extended to a network of frontal, temporal and motor-sensory regions at the voxel level. A similar observation has been reported by Oechslin et al. (2013), where larger hippocampal volume was associated with better general cognitive abilities in younger musicians (professionals and amateurs), but not in non-musicians. The current study highlights that a similar association is detectable in OA with musical experience, where it is linked to distributed brain areas supporting sensory, motor and cognitive functions.

In general, the present result may reflect a more efficient use of an overall younger brain age in musically active people, as shown previously (Rogenmoser et al., 2018). Interestingly, the observed frontal-to-temporal regions partially overlap with brain networks that show enhanced functional and/or structural connectivity in young to middle-aged musicians compared to non-musicians (Halwani et al., 2011; Andrews et al., 2021; Leipold et al., 2021) and support cognitive reserve processes in older age (Colangeli et al., 2016; Marques et al., 2016; Franzmeier et al., 2017; Benson et al., 2018). In this light, our findings may imply that the long-term playing of a musical instrument could be associated with a more efficient recruitment of dedicated brain networks, as a potential benefit that might help to preserve cognitive health in late life.

### Musical Activity and Brain Morphology

In our study, musical activity was not significantly associated with greater brain reserve in higher-order brain regions. More precisely, participants with musical activity did not differ from controls in GMV of the pre-selected frontal, temporal or hippocampal regions. Our voxel-based analysis largely confirmed the ROI-based observations. Merely a smaller cluster with larger GMV was found in the somatosensory area of participants with musical activity compared to controls, presumably reflecting brain plasticity in response to the intense tactile stimulations induced by playing a musical instrument (Gaser and Schlaug, 2003; Gärtner et al., 2013). Earlier studies have shown a positive association between musical activity and GMV within higher-order fontal, temporal, and also parietal regions, albeit mainly in young musician compared to controls (Gaser and Schlaug, 2003; Groussard et al., 2010; Gärtner et al., 2013) with limited evidence in OA (Chaddock-Heyman et al., 2021).

Importantly, we accounted for several reserve proxies that may help to preserve brain morphology in late life (Arenaza-Urquijo et al., 2013; Wirth et al., 2014) and thereby act as potential confounders. Given this effort, it seems reasonable to assume that there is a limited added benefit of musical activity on structural brain resources (as measured using GMV) in OA within regions that are susceptible to aging and AD. Alternatively though, subtle morphological associations of musical activity could be unnoticed in the older population, due to increased morphological variability by brain aging and/or brain pathology as found in a considerable proportion of cognitively unimpaired OA (Hedden and Gabrieli, 2004; Knopman et al., 2012; Wirth et al., 2013). Lastly, it is important to note that previous cross-sectional studies reporting a positive association between musical activity and GMV in hippocampal regions as well as WM microstructural integrity included professional experts with intense musical/auditory experience (Groussard et al., 2010; Teki et al., 2012; Andrews et al., 2021). Therefore, the lack of current results with regard to a presumed modification of GMV through long-term musical activity may also have to do with the different proficiency level of our cohort.

### Synopsis and Concluding Remarks

Taken together, the present study adds supportive evidence to the picture that participation in musical activity may constitute a protective factor in OA. Nevertheless, the observed health benefits associated with playing a musical instrument could be encouraged by a general engagement in an advantageous lifestyle. Those participants reporting musical activity were characterized by a high-reserve profile, including higher education, SES, crystallized intelligence, and more frequent participation in physical activity. A similar pattern was observed in previous studies (Mansens et al., 2018), but not in all (Hanna-Pladdy and Gajewski, 2012; Gray and Gow, 2020). Together these variables may resemble a lifestyle that comprises various beneficial body and mind activities that could in synergy be associated with cognitive or brain reserve in late life (Kempermann, 2019b,^2022^). Notably though, we observed superior cognitive abilities in the musically active group with those reserve proxies accounted for by our one-to-one matching procedure. This may suggest an added benefit of musical activity on late-life brain and cognitive functions. In addition, more emphasis could be placed on the assessment of a more holistic lifestyle (i.e., going beyond individual lifestyle activities) to highlight associations and presumed synergies with brain aging and reserve in future studies.

One might argue that high-functioning individuals are more likely to engage in and continue to play a musical instrument during life. In line with this argumentation, one might further expect that these high performers exhibit higher education, intelligence, and SES compared to controls. Indeed, there is confirmatory evidence to suggest that greater early-adulthood general cognitive abilities predict educational and occupational success in later adulthood (Daly et al., 2015; Kremen et al., 2019). In the present study, however, the high-functioning group was comparable to controls in the above-mentioned measures, which were deliberately accounted for when selecting the well-matched control group. Given this notion, one may reason that reverse causation seems less likely to apply to our findings, with caution that needs to be considered in correlational findings (Schellenberg, 2020).

Overall, our results converge with the view that musical activity may serve as a low-threshold multimodal enrichment strategy throughout life until old age. However, targeted intervention studies are needed to validate the impact of musical activity on late-life cognitive abilities and underlying brain correlates in OA (James et al., 2020). In light of our findings, it may be proposed that musical activity and the associated mulitmodal stimulations could strengthen cognitive resilience through benefits involving neural capacities and connections in dedicated motor-sensory-cognitive brain networks, as suggested by previous studies (Halwani et al., 2011; Andrews et al., 2021; Leipold et al., 2021). Prospective longitudinal and interventional studies must clarify whether or not musically active older people are indeed more protected against cognitive decline, which could inform targeted public health strategies.

### Strengths and Limitations

Our study has several strengths. We assembled data from the observational DELCODE cohort to assess a well-characterized sample of cognitively unimpaired OA with and without self-reported participation in musical activity using cognitive, behavioral, and brain volume measures. All measures were acquired in the same participants using standardized operation procedures and high-quality data assessments. The detailed phenotyping, as provided by cohort-based studies, generated new evidence on potential brain and cognitive health benefits associated with musical activity in the older population. Moreover, we examined late-life cognitive abilities using latent factor scores, which can be generalized above the measurement method. Lastly, the availability of a wide range of variables made it possible to account for several reserve proxies, known to be enhanced in musically active older people (e.g., Mansens et al., 2018).

Several limitations need to be considered. (1) Our cohort-based approach included a limited description of musical activity/experience in the present older participants. More detailed information on lifetime musical activity including, e.g., the type of musical instrument, age of acquisition, training intensity and other musical abilities would be desirable (Okely et al., 2021), given that these features may impact brain plasticity and cognitive abilities (Bangert and Schlaug, 2006; Hanna-Pladdy and MacKay, 2011). (2) Due to the cross-sectional design, caution is needed in drawing conclusions on the directionality of the here-observed associations. Although we accounted for a number of confounding variables, it may be possible that other unmeasured variables facilitate playing a musical instrument across the life course, such as genetic predispositions, personality traits or early-life environmental exposures (Corrigall et al., 2013; Zatorre, 2013; Altenmüller and Furuya, 2017). These factors could play a role in the observed relationships, warranting further investigation. (3) The assessment of musical activity was based on self-reports that were partially retrospective. The information was extracted from the LEQ, as a validated questionnaire that has been applied in the assessment of cognitive reserve/resilience (Chan et al., 2018; Collins et al., 2021). Self-reports can be biased by the current cognitive status of a person. However, our participants were cognitively unimpaired and screened for current and passed mental health conditions. Based on our study, it can be recommended that cohort studies include a more detailed and objective evaluation of lifetime musical activity (e.g., Okely et al., 2021) to strengthen validity and accuracy of the measure. (4) Finally, it is important to note that other aspects of musical activity, such as listening to music or choir singing, have beneficial effects in healthy and cognitively impaired OA, e.g., in the rehabilitation or intervention of neurological and neurodegenerative conditions (Särkämö, 2018; Gold et al., 2019).

## Conclusion

To conclude, the present findings are promising to suggest that long-term participation in musical activity, as an accessible leisure-time activity, could be associated with greater brain and cognitive health in late life. Well-designed studies in OA are needed to further assess detailed information about the nature of playing a musical instrument and underlying functional and structural brain correlates associated with this complex multimodal activity.

## Data Availability Statement

The data that support findings of the present study are available on reasonable request from the DELCODE database. Requests to access these datasets should be directed to the German Center for Neurodegenerative Diseases (DZNE), Bonn.

## Ethics Statement

The general study protocol for the DELCODE study was approved by the ethical committees of the medical faculties of all sites, i.e., the ethical committees of Berlin (Charité – Universitätsmedizin), Bonn (Medical Faculty, University of Bonn), Cologne (Medical Faculty, University of Cologne), Göttingen (Universitätsmedizin Göttingen), Magdeburg (Medical Faculty, Otto-von-Guericke University, Magdeburg), Munich (Medical Faculty, Ludwig-Maximilians-Universität), Rostock (Medical Faculty, University of Rostock), and Tübingen (Medical Faculty, University of Tübingen). The process was led and coordinated by the ethical committee of the medical faculty of the University of Bonn under the registration number: 171/13. The patients/participants provided written informed consent to participate in the DELCODE study.

## Author Contributions

AB, TK, AH, SR, MWa, GK, and MWi: conceptualization and design of the current study. OP, SF, JP, SA, ASc, IF, ASp, NR, SW, LK, JW, CB, FM, CM, LD, RY, KB, DJ, RP, B-SR, ST, IK, CL, MM, JH, PD, ME, KS, ED, FJ, SR, and MWa: overall design and implementation of the DELCODE study. AB, AZ, MG, and MW: methodology and statistical analysis. AB, AZ, TK, AH, SW, SR, MWa, GK, and MWi: interpretation of data. AB, AZ, TK, MG, AH, OP, SF, JP, SA, ASc, KF, IF, ASp, NR, SW, LK, JW, CB, FM, CM, LD, RY, KB, DJ, RP, B-SR, ST, IK, CL, MM, JH, PD, ME, KS, ED, FJ, SR, MWa, GK, and MWi: drafting and/or revision of manuscript. All authors contributed to the article and approved the submitted version.

## Conflict of Interest

OP received fees for consultation from Abbvie, Biogen, Eisai, Griffols, MSD Roche, and Schwabe. JP received fees for consultation, lectures, and patents from Neurimmune, Axon, Desitin, and Epomedics. JW was an advisory board member of Abbott, Biogen, Boehringer Ingelheim, Immunogenetics, Lilly, MSD Sharp & Dohme, and Roche Pharma and received honoraria for lectures from Actelion, Amgen, Beeijing Yibai Science and Technology Ltd., Janssen Cilag, Med Update GmbH, Pfizer, Roche Pharma and holds the following patents: PCT/EP 2011 001724 and PCT/EP 2015 052945. JW was supported by an Ilidio Pinho professorship, iBiMED (UIDB/04501/2020) at the University of Aveiro, Portugal. ED received fees for consultation from Roche, Biogen, RoxHealth and holds shares in neotiv. FJ received fees for consultation from Eli Lilly, Novartis, Roche, BioGene, MSD, Piramal, Janssen, and Lundbeck. The remaining authors declare that the research was conducted in the absence of any commercial or financial relationships that could be construed as a potential conflict of interest.

## Publisher’s Note

All claims expressed in this article are solely those of the authors and do not necessarily represent those of their affiliated organizations, or those of the publisher, the editors and the reviewers. Any product that may be evaluated in this article, or claim that may be made by its manufacturer, is not guaranteed or endorsed by the publisher.

## Footnotes

1
http://surfer.nmr.mgh.harvard.edu/

2
http://dbm.neuro.uni-jena.de/vbm

3
https://github.com/bioimagesuiteweb/bisweb

4
http://marsbar.sourceforge.net/
