# Supplementary material for: Musical Activity During Life Is Associated With Multi-Domain Cognitive and Brain Benefits in Older Adults
Source: Front Psychol. 2022 Aug 25;13:945709. doi: 10.3389/fpsyg.2022.945709 (PMC9454948; doi:10.3389/fpsyg.2022.945709)
Supplement: Supplementary file 1 [file Data_Sheet_1.docx]

## Musical activity during life is associated with multi-domain cognitive and brain benefits in older adults

# Supplementary methods

## Overall design of the DELCODE study

The data used in the present study were obtained from the DELCODE study. The detailed study protocol can be found in the previous protocol report (Jessen et al., 2018). In brief, the DELCODE cohort was set up to recruit 1000 participants at baseline with five groups of participants. Specifically, these groups are older adults (OA), first-degree relatives of AD patients (family history, FH) as well as participants with subjective cognitive decline (SCD), mild cognitive impairment (MCI), and mild AD dementia. At baseline assessment, all participants received extensive clinical, neuropsychological, and behavioral assessments. To minimize site-effects and ensure high data quality, assessment protocols were standardized across sites using Standard Operating Procedures (SOP). Post-scanning MRI image quality assessments were conducted by the DZNE Magdeburg. The DELCODE study protocol agreed with ethical principles for human experimentation in accordance with the Declaration of Helsinki. At each participating study sites, the protocol was approved by the local ethical committees. All participants gave their written informed consent. DELCODE was registered at the German Clinical Trials Register (DRKS00007966; date: 2015/05/04).

## Participant selection

The DELCODE baseline dataset (total: *n* = 1079, data release for this study: 01.2021) was used to select a subset of participants into the present study (**supplementary Figure S1**): A total of 943 participants had structural cranial magnetic resonance imaging (MRI) assessments. Of these participants, cognitively unimpaired participants were selected (i.e., OA, SCD, FH, total: *n* = 678). Subsequently, participants with a self-reported participation in musical activity across the life course (group of interest9 and participants without musical activity (control group) were identified (total: *n* = 429), using the Lifetime of Experiences Questionnaire (LEQ, Valenzuela and Sachdev, 2007). In the last step, we selected a well-matched control group including only participants with complete datasets, resulting in a final sample of *n* = 140 participants with *n* = 70 participants in each musical activity group.

###
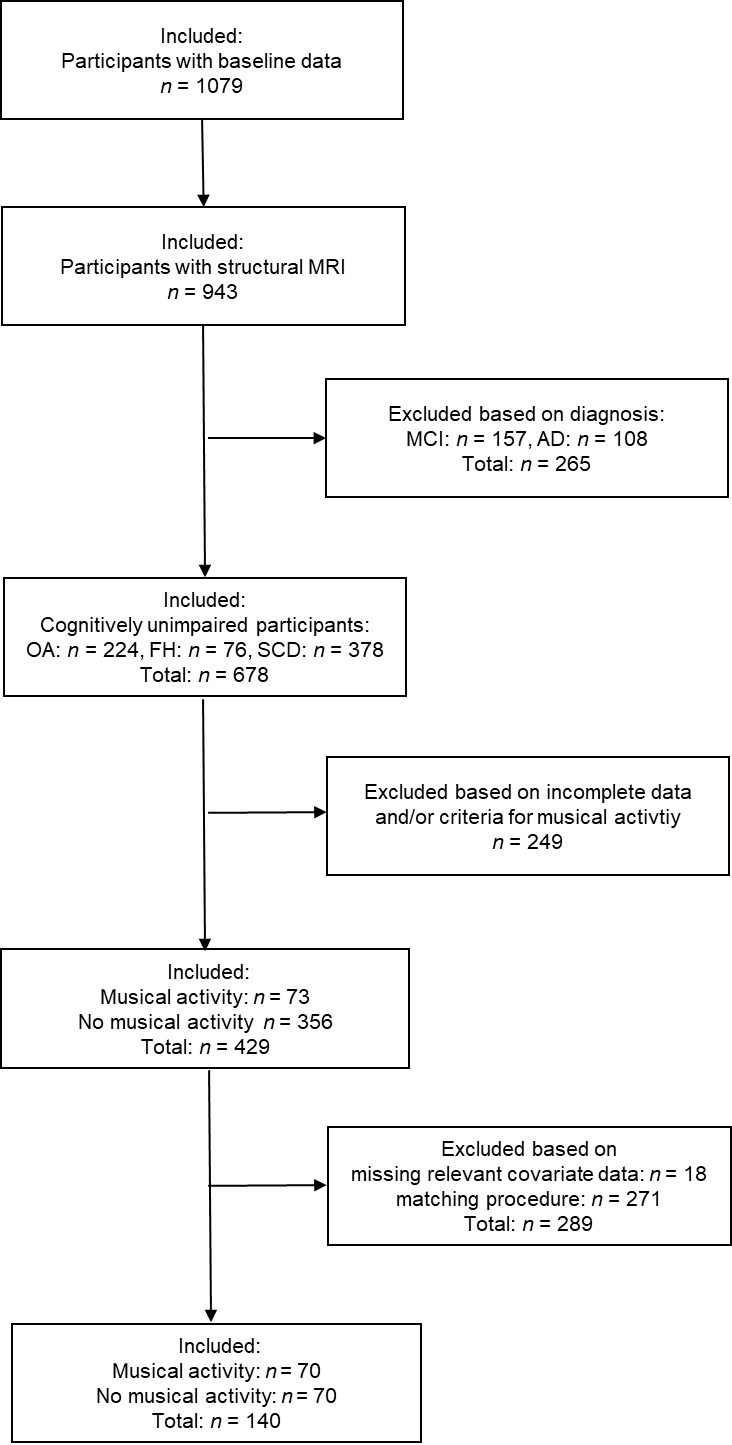
Figure S1

**Figure S1: Participant selection flowchart.** ***Key:*** AD, Alzheimer’s disease; FH, family history of AD; MCI, mild cognitive impairment; MRI, Magnetic resonance imaging; OA, older adults; SCD, subjective cognitive decline.

## Measurement of musical activity

Self-reported participation in musical activity across the life course was assessed using the LEQ (Valenzuela and Sachdev, 2007) adapted for the German population (Roeske et al., 2018). This validated questionnaire measures educational, occupational, and cognitive lifestyle activities over three life periods (young adulthood: 13 – 30 years, mid-life: 30 – 65 years, and late-life: 65 years onwards). Within each life period, several complex activities are assessed including educational attainment, occupational history and participation in multiple leisure activities including (1) playing a musical instrument, (2) social outings, (3) artistic activity (drawing, painting, writing), (4) physical activity, (5) reading, and (6) speaking a second language. The frequency of musical activity was measured across the life periods using the respective LEQ item (‘How often did you play an instrument?‘) with responses provided on a 6-point Likert scale (0/‘never‘; 1/‘less than 1 time per month‘, 2/‘1 time per month‘, 3/‘2 times per month‘, 4/‘weekly‘, 5/‘daily‘).

Using a coding scheme, we evaluated musical activity as a binary groups variable: (1) The group with musical activity during life (group of interest) included only participants that were musically active by playing a musical instrument in all given life periods (i.e., no life period included ‘never’). This sample comprised *n* = 70 participants with musical activity during life and was characterized descriptively using the individual trajectories of musical activity across the given life periods (**supplementary Figure S2**). All participants reported higher frequency of musical instrument playing (‘2 times per month’ or more) in at least one life period and otherwise lower frequency of musical activity (less than ‘2 times per month’). (2) The no musical activity group (control group) included *n* = 70 well-matched participants that reported to never have played a musical instrument in any of the given life periods (data not shown). To ensure that there were no false classifications, participants with missing responses on the musical activity item in any life period were excluded.

### Figure S2


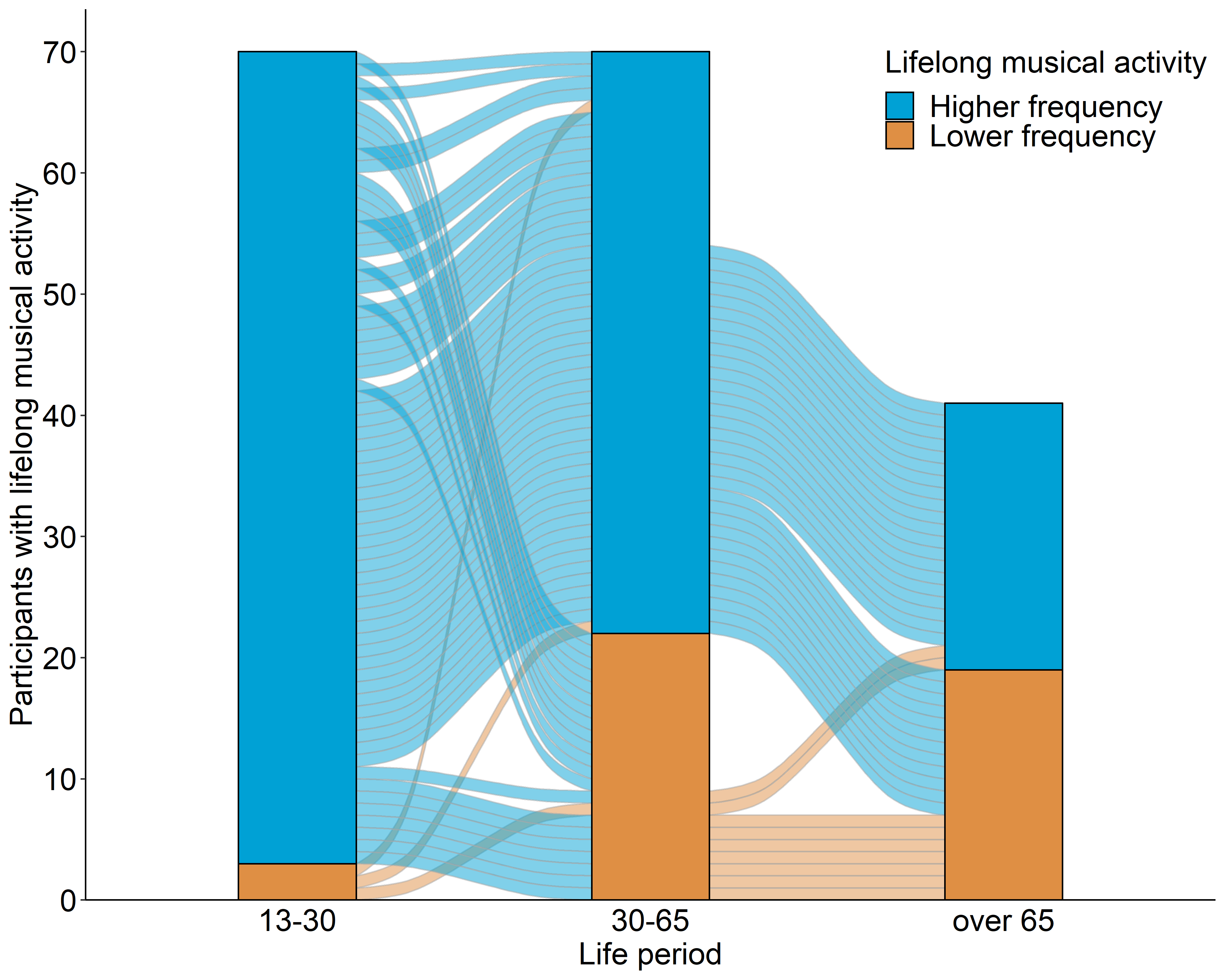


**Figure S2: Descriptive characterization of participants with self-reported participation in musical activity across the three life periods.** The alluvial plot shows the individual trajectories of those DELCODE participants (n = 70) that reported playing a musical instrument across the life course, including young adulthood (13 – 30 years), mid-life (30 – 65 years), and late-life (65 years onwards) if applicable. All participants reported musical activity with higher or lower frequency of participation. Higher frequency of musical activity corresponded to ‘2 times per month’ or more and lower frequency corresponded to less than ‘2 times per month’. Note, depending on a person’s age, the assessment of musical activity was based on two (< 65 years of age) or three (≥ 65 years of age) life periods.

## Measurement of cognitive abilities

Multi-domain cognitive abilities were assessed by five cognitive composite scores extracted from the extensive neuropsychological test battery of the DELCODE study using confirmatory factor analysis described in the previous study (Wolfsgruber et al., 2020). In brief, Wolfsgruber and colleagues (2020) extracted a latent five-factor structure using confirmatory factor analyses (CFA) with robust maximum likelihood (MLR) estimation. Variance and mean of the latent factors were fixed to one and zero, respectively. The assignment of indicator variables to the latent factors was motivated by previous CFAs using similar test batteries and similar cohort studies (Dowling et al., 2010;Park et al., 2012). Factor score estimates of the latent variables were extracted using the multivariate regression method (Grice, 2001). In addition, a global cognitive score was created by taking the mean of the five extracted domain factor scores (Wolfsgruber et al., 2020). A graphical representation of the five latent factors can be found in the supplement of Wolfsgruber and colleagues (2020). Inter-correlations between the cognitive composite scores in the present sample ranged from ~0.6 – 0.8 (data not shown), as reported previously (Wolfsgruber et al., 2020).

## Measurement of socioeconomic status

The socioeconomic status (SES) was calculated for each participant using the international socio-economic index of occupational information score (ISEI, min. score: 16, max. score: 90) (Ganzeboom et al., 1992). The measure was estimated using the occupational history of each participant, as assessed by the LEQ. In brief, occupational activities of each participant were obtained across 10 five-year intervals from middle-to-late adulthood (age 30 to 79 years) and coded into occupational categories using the O*Net code system (<https://www.onetonline.org/>) (Peterson et al., 2001). Next, the O*Net codes were converted into ISEI scores using fully-automated publicly-available crosswalk procedures that included conversion to Standard Occupational Classification codes (SOC), International Standard Classification of Occupations (ISCO-08) and final ISEI scores calculation (<http://www.harryganzeboom.nl/ISCO08/index.htm>, retrieved: 2021/04). The ISEI scores were averaged across time intervals to obtain one mean SES measure per participant. The SES measure was positively and significantly associated with the LEQ sum scores measuring educational as well as occupational activity for young (*n* = 140, *r* = 0.54, *p* < 0.001) and middle (*n* = 140, *r* = 0.69, *p* < 0.001) adulthood, indicating the validity of the estimated ISEI scores.

## Construction of Regions-of-interest (ROIs)

The frontal and temporal ROI were calculated following the procedure previously proposed by Desikan and colleagues (Desikan et al., 2006). The frontal ROI was calculated as the sum over left and right hemisphere comprising the superior frontal gyrus, middle frontal gyrus (rostral and caudal division), inferior frontal gyrus (pars opercularis, pars triangularis and pars orbitalis), orbitofrontal cortex (lateral and medial division), frontal pole, precentral gyrus and the paracentral lobule. The temporal ROI was calculated as the sum over left and right hemisphere comprising the medial temporal lobe (entorhinal cortex, parahippocampal gyrus, temporal pole and fusiform gyrus) and the lateral temporal lobe (superior temporal gyrus, middle temporal gyrus, inferior temporal gyrus, transverse temporal cortex and the banks of the superior temporal sulcus).

## Propensity matching and pre-matching sample

Based on an existing large-scale population-based study (Mansens et al., 2018), we expected differences in known reserve proxies between the two musical activity groups. Pre-analytical comparisons of DELCODE participants with musical activity (*n* = 73) and controls (*n* = 356) confirmed the presence of higher educational attainment, crystallized intelligence, SES, and both long-term and current physical activity in participants with musical activity (all *p’s* < 0.05, **supplementary Table S1**). We applied propensity score matching based on relevant covariates with the goal to identify a well-balanced control group (Ho et al., 2011;Zhang, 2017). This well-matched control group was identified taking into account age, sex, diagnostic category, education, crystallized intelligence, SES and long-term physical activity.

==================

### Table S1: Descriptive characteristics of the pre-matching DELCODE sample (n = 429)

|  | Musical activity | No musical activity | P value |
| --- | --- | --- | --- |
| Number (n) | 73 | 356 | - |
| Age (years) | 68.22 (6.60) | 69.44 (5.82) | 0.146 |
| Gender female/male (n) | 33/40 | 192/164 | 0.174 |
| Education (years) | 16.11 (2.75) | 14.28 (2.83) | < 0.001*** |
| Diagnostic group OA/FH/SCD (n) | 19/7/47 | 129/35/192 | 0.221 |
| SES ^a^ | 65.48 (16.82), *n* = 72 | 59.33 (17.64), *n* = 347 | 0.006** |
| Crystalized intelligence ^b^ | 33.29 (2.19), *n* = 72 | 32.08 (2.56), *n* = 353 | < 0.001*** |
| Physical activity, long-term ^c^ | 4.25 (0.77), *n* = 72 | 3.73 (1.13), *n* = 353 | < 0.001*** |
| Physical activity, current ^d^ | 33.94 (11.58), *n* = 69 | 30.60 (12.32), *n* = 346 | 0.033* |
| Descriptive data are given if applicable as mean and standard deviation (in parenthesis). The actual sample size is provided, if different from sample size specified in first row. *P*-values correspond to independent *t*-tests for unequal variance with participant group as independent variable. Chi-square statistic was used to compare the distribution of categorical variables.  ****p* < 0.001, ***p* < 0.01, **p* < 0.05.  ***Key:*** HC, healthy control participants; FH, participants with a family history of AD; GMV, gray matter volume; SCD, participants with subjective cognitive decline; SES, socioeconomic status.  ^a^ International socio-economic index (ISEI); ^b^ Multiple-Choice Vocabulary Intelligence Test (MWT); ^c^ Lifetime of Experiences Questionnaire (LEQ); ^d^ Physical Activity Scale for the Elderly (PASE). | | | |

==================

# Supplementary results

### Table S2: Linear regression analysis between musical activity and GMV in regions-of-interest

|  | **Dependent variable** | | **Independent variable** | **B** | **SE B** | **Beta** | **P value** | **Total R^2^ (adj.)** |
| --- | --- | --- | --- | --- | --- | --- | --- | --- |
|  | **ROI** | **Hemisphere** |  |  |  |  |  |  |
| 1 | Frontal GMV | Left | Musical Activity | -0.026 | 0.104 | -0.021 | 0.807 | 0.120 (0.052) |
| 2 | Frontal GMV | Right | Musical Activity | -0.020 | 0.101 | -0.018 | 0.839 | 0.120 (0.052) |
| 3 | Temporal GMV | Left | Musical Activity | -0.021 | 0.071 | -0.025 | 0.769 | 0.121 (0.053) |
| 4 | Temporal GMV | Right | Musical Activity | -0.032 | 0.073 | -0.038 | 0.663 | 0.122 (0.053) |
| 5 | Hpc GMV | Left | Musical Activity | 0.000 | 0.000 | -0.053 | 0.549 | 0.095 (0.024) |
| 6 | Hpc GMV | Right | Musical Activity | 0.000 | 0.000 | -0.050 | 0.564 | 0.110 (0.041) |
| Models adjusted for scanner site.  Musical activity was included as binary predictor, dummy coded with musical activity = 1, no musical activity = 0.  Regional GMV was adjusted by total intracranial volume (TIV).  ***Key*:** B, unstandardized coefficient; Hpc, Hippocampus; SE, standard error; Beta, standardized coefficient; R^2^, explained variance; GMV, gray matter volume. | | | | | | | | |

==================

### Table S3: Interaction analysis between musical activity and temporal GMV

|  | **Dependent variable** | **Independent variable** | **B** | **SE B** | **Beta** | **P value** | **Total R^2^ (adj.)** |
| --- | --- | --- | --- | --- | --- | --- | --- |
| 1 | Global cognition | Music Activity × temporal GMV | 0.403 | 0.198 | 0.241 | 0.044* | 0.364 (0.304) |
| 2 | Learning and Memory | Music Activity × temporal GMV | 0.179 | 0.188 | 0.118 | 0.345 | 0.293 (0.226) |
| 3 | Working Memory | Music Activity × temporal GMV | 0.507 | 0.206 | 0.298 | 0.015* | 0.336 (0.273) |
| 4 | Executive Functions | Music Activity × temporal GMV | 0.347 | 0.207 | 0.207 | 0.097 | 0.307 (0.241) |
| 5 | Language | Music Activity × temporal GMV | 0.400 | 0.190 | 0.253 | 0.038* | 0.342 (0.280) |
| 6 | Visuospatial | Music Activity × temporal GMV | 0.290 | 0.206 | 0.177 | 0.162 | 0.287 (0.220) |
| Models adjusted for scanner site.  Musical activity was included as binary predictor, dummy coded with musical activity = 1, no musical activity = 0.  Temporal GMV was adjusted for total intracranial volume and mean centered.  **p* < 0.05.  ***Key:*** B, unstandardized coefficient; SE, standard error; Beta, standardized coefficient; R^2^, explained variance. | | | | | | | |

==================

### Table S4: Interaction analysis between musical activity and hippocampal GMV

|  | **Dependent variable** | **Independent variable** | **B** | **SE B** | **Beta** | **P value** | **Total R^2^ (adj.)** |
| --- | --- | --- | --- | --- | --- | --- | --- |
| 1 | Global cognition | Music Activity × Hpc GMV | 2.248 | 2.515 | 0.118 | 0.373 | 0.330 (0.266) |
| 2 | Learning and Memory | Music Activity × Hpc GMV | -0.727 | 2.350 | -0.042 | 0.757 | 0.284 (0.216) |
| 3 | Working Memory | Music Activity × Hpc GMV | 4.109 | 2.620 | 0.211 | 0.119 | 0.299 (0.233) |
| 4 | Executive Functions | Music Activity × Hpc GMV | 2.268 | 2.640 | 0.118 | 0.392 | 0.268 (0.199) |
| 5 | Language | Music Activity × Hpc GMV | 2.782 | 2.435 | 0.154 | 0.255 | 0.300 (0.234) |
| 6 | Visuospatial | Music Activity × Hpc GMV | 0.897 | 2.547 | 0.047 | 0.725 | 0.290 (0.223) |
| Models adjusted for scanner site.  Musical activity was included as binary predictor, dummy coded with musical activity = 1, no musical activity = 0.  Hippocampal GMV was adjusted for total intracranial volume and mean centered.  ***Key:*** B, unstandardized coefficient; Hpc, hippocampus; SE, standard error; Beta, standardized coefficient; R^2^, explained variance. | | | | | | | |

==================

### Table S5: Analysis between musical activity and GMV at the voxel level

| **No. cluster** | **Label** | **Brodmann area (BA)** | **Hemisphere** | **Cluster** | | **Peak of cluster** | | | |
| --- | --- | --- | --- | --- | --- | --- | --- | --- | --- |
|  |  |  |  | ***p*** | **size** | **Z value** | **MNI coordinates**  **(x y z)** | | |
| **Main effect ^a^** | | | | | | | | | |
| 1 | Postcentral gyrus | BA 6 | left | 0.195 | 220 | 3.60 | -57 | -6 | 27 |
| **Interaction effect: GLOBAL ^b^** | | | | | | | | | |
| 1 | Inferior middle temporal gyrus | BA 20 | right | 0.023 | 730 | 4.57 | 50 | -14 | -42 |
| 2 | Precentral gyrus | BA 6 | left | 0.051 | 509 | 4.17 | -40 | -6 | 52 |
| 3 | Postcentral gyrus | BA 1 | right | 0.240 | 170 | 3.74 | 50 | -18 | 60 |
| **Interaction effect: MEM ^c^** | | | | | | | | | |
| 1 | Middle frontal gyrus | BA 6 | left | 0.210 | 198 | 3.60 | -40 | -2 | 50 |
| **Interaction effect: WM ^d^** | | | | | | | | | |
| 1 | Precentral gyrus † | BA 6 | left | 0.025 | 718 | 4.90 | -28 | -10 | 48 |
| 2 | Precentral gyrus | BA 4 | left | 0.114 | 322 | 4.81 | -39 | -14 | 33 |
| 3 | Inferior middle temporal gyrus | BA 20 | right | 0.054 | 502 | 4.45 | 51 | -12 | -42 |
| 4 | Fusiform gyrus | BA 20 | left | 0.279 | 145 | 3.99 | -36 | -8 | -38 |
| 5 | Inferior frontal gyrus | BA 46 | right/  lateral | 0.140 | 277 | 3.90 | 40 | 33 | 12 |
| 6 | Anterior medial temporal lobe | BA 38 | left | 0.245 | 168 | 3.78 | -38 | 16 | -36 |
| 7 | Orbito-frontal gyrus | BA 11 | medial/right | 0.166 | 242 | 3.59 | 6 | 36 | -14 |
| 8 | Postcentral gyrus | BA 4 | right | 0.293 | 137 | 3.52 | 50 | -8 | 26 |
| **Interaction effect: EXEC ^e^** | | | | | | | | | |
| 1 | Inferior middle temporal gyrus † | BA 20 | right | 0.005 | 1193 | 5.39 | 51 | -12 | -42 |
| 2 | Fusiform gyrus | BA 20 | left | 0.114 | 318 | 4.22 | -36 | -6 | -38 |
| 3 | Hippocampus | Hippocampus | right | 0.056 | 484 | 4.09 | 36 | -16 | -16 |
| 4 | Anterior medial temporal lobe | BA 38 | left | 0.288 | 138 | 3.70 | -36 | 9 | -38 |
| 5 | Orbito-frontal gyrus | BA 11 | medial/right | 0.215 | 189 | 3.56 | 8 | 36 | -15 |
| 6 | Postcentral gyrus | BA 1 | right | 0.296 | 133 | 3.49 | 51 | -20 | 54 |
| **Interaction effect: LAN ^f^** | | | | | | | | | |
| 1 | Inferior middle temporal gyrus | BA 20 | right | 0.111 | 326 | 3.99 | 51 | -12 | -42 |
| **Interaction effect: VIS ^g^** | | | | | | | | | |
| 1 | Fusiform gyrus | BA 36 | right | 0.195 | 213 | 4.13 | 34 | -14 | -34 |
| 2 | Precentral gyrus | BA 6 | left | 0.083 | 399 | 4.11 | -30 | -10 | 51 |
| 3 | Inferior middle temporal gyrus | BA 20 | right | 0.210 | 198 | 3.95 | 48 | -14 | -42 |
| Models adjusted for scanner site and TIV.  Musical activity was included as binary predictor, dummy coded with musical activity = 1, no musical activity = 0.  ^a^ Results from the main effect model with musical activity and GMV (*p* < 0.001 uncorrected, expected voxels per cluster k = 140).  ^b^ Results from the interaction effect model with musical activity, global cognition, and GMV (*p* < 0.001 uncorrected, expected voxels per cluster *k* = 133).  ^c^ Results from the interaction effect model with musical activity, learning and memory, and GMV (*p* < 0.001 uncorrected, expected voxels per cluster *k* = 135).  ^d^ Results from the interaction effect model with musical activity, working memory, and GMV (*p* < 0.001 uncorrected, expected voxels per cluster *k* = 134).  ^e^ Results from the interaction effect model with musical activity, executive function, and GMV (*p* < 0.001 uncorrected, expected voxels per cluster *k* = 132).  ^f^ Results from the interaction effect model with musical activity, executive function, and GMV (*p* < 0.001 uncorrected, expected voxels per cluster *k* = 133).  ^g^ Results from the interaction effect model with musical activity, executive function, and GMV (*p* < 0.001 uncorrected, expected voxels per cluster *k* = 136).  † Significant after FWE correction (*p* < 0.05, expected voxels per cluster *k* = 42 both for WM and EXEC).  Cluster peaks are specified by their anatomical site, labelled using the Hammersmith atlas provided by the CAT12 toolbox.  Brodmann areas were identified with the BioImage Suite Web 1.2.0.  ***Key:*** GMV, gray matter volume; MNI coordinates (x y z), coordinates in millimeters; TIV, total intracranial volume; GLOBAL, global cognition; MEM, learning and memory; WM, working memory; EXEC, executive function; LAN, language abilities; VIS, visuospatial abilities. | | | | | | | | | |

==================

### Figure S3

**
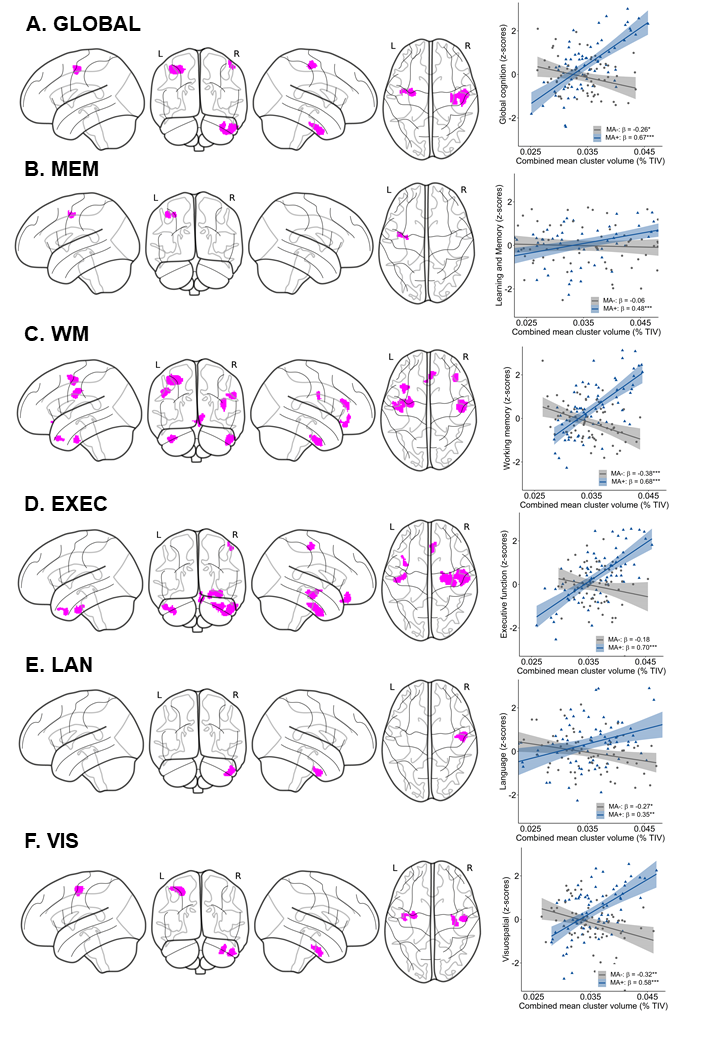
**

**Figure S3:** **Associations between musical activity and regional volume distribution.** **A-F.** **Results of the moderation analysis**. The statistical maps display clusters (*p* < .001 uncorrected, color-coded in magenta) with a significant moderation effect of musical activity for global cognition (A, GLOBAL), learning and memory (B, MEM), working memory (C, WM), executive functions (D, EXEC), language (E, LAN), and visuospatial abilities (F, VIS). Corresponding scatter plots show the associations using mean values extracted from the GMV maps in the combined cluster. Larger GMV in the combined cluster was associated with better cognitive abilities selectively in musically active participants (MA+, blue) compared to controls (MA-, gray). Individual data points, linear trends (solid lines), 95% confidence intervals (shaded areas), and standardized regression coefficients (β) within each musical activity group are provided. Significance levels (uncorrected): ****p* < 0.001, ***p* < 0.01, **p* < 0.05. ***Key:*** GMV, gray matter volume; TIV, total intracranial volume.

==================

# References

Desikan, R.S., Segonne, F., Fischl, B., Quinn, B.T., Dickerson, B.C., Blacker, D., Buckner, R.L., Dale, A.M., Maguire, R.P., Hyman, B.T., Albert, M.S., and Killiany, R.J. (2006). An automated labeling system for subdividing the human cerebral cortex on MRI scans into gyral based regions of interest. *NeuroImage* 31**,** 968-980.

Dowling, N.M., Hermann, B., La Rue, A., and Sager, M.A. (2010). Latent structure and factorial invariance of a neuropsychological test battery for the study of preclinical Alzheimer's disease. *Neuropsychology* 24**,** 742-756.

Ganzeboom, H.B.G., De Graaf, P.M., and Treiman, D.J. (1992). A standard international socio-economic index of occupational status. *Social Science Research* 21**,** 1-56.

Grice, J.W. (2001). Computing and evaluating factor scores. *Psychol Methods* 6**,** 430-450.

Ho, D., Imai, K., King, G., and Stuart, E.A. (2011). MatchIt: Nonparametric Preprocessing for Parametric Causal Inference. *Journal of Statistical Software* 42**,** 1 - 28.

Jessen, F., Spottke, A., Boecker, H., Brosseron, F., Buerger, K., Catak, C., Fliessbach, K., Franke, C., Fuentes, M., Heneka, M.T., Janowitz, D., Kilimann, I., Laske, C., Menne, F., Nestor, P., Peters, O., Priller, J., Pross, V., Ramirez, A., Schneider, A., Speck, O., Spruth, E.J., Teipel, S., Vukovich, R., Westerteicher, C., Wiltfang, J., Wolfsgruber, S., Wagner, M., and Duzel, E. (2018). Design and first baseline data of the DZNE multicenter observational study on predementia Alzheimer's disease (DELCODE). *Alzheimers Res Ther* 10**,** 15.

Mansens, D., Deeg, D.J.H., and Comijs, H.C. (2018). The association between singing and/or playing a musical instrument and cognitive functions in older adults. *Aging Ment Health* 22**,** 964-971.

Park, L.Q., Gross, A.L., Mclaren, D.G., Pa, J., Johnson, J.K., Mitchell, M., and Manly, J.J. (2012). Confirmatory factor analysis of the ADNI Neuropsychological Battery. *Brain Imaging Behav* 6**,** 528-539.

Peterson, N., Mumford, M., Borman, W., Jeanneret, P., Fleishman, E., Levin, K., Campion, M., Mayfield, M., Morgeson, F., Pearlman, K., Gowing, M., Lancaster, A., Silver, M., and Dye, D. (2001). Understanding Work Using the Occupational Information Network (O*NET): Implications for Practice and Research. *Personnel Psychology* 54**,** 451-492.

Roeske, S., Wolfsgruber, S., Kleineidam, L., Zulka, L., Buerger, K., Ewers, M., Laske, C., Nestor, P., Peters, O., Priller, J., Schneider, A., Spottke, A., Ramirez, A., Heneka, M., Teipel, S.J., Wiltfang, J., Okonkwo, O.C., Kalbe, E., Düzel, E., Jessen, F., Wagner, M., and Group, D.S. (2018). P3-591: A German version of the lifetime of experiences questionnaire (LEQ) to measure cognitive reserve: Validation results from the DELCODE study. *Alzheimer's & Dementia* 14**,** P1352-P1353.

Valenzuela, M.J., and Sachdev, P. (2007). Assessment of complex mental activity across the lifespan: development of the Lifetime of Experiences Questionnaire (LEQ). *Psychological medicine* 37**,** 1015-1025.

Wolfsgruber, S., Kleineidam, L., Guski, J., Polcher, A., Frommann, I., Roeske, S., Spruth, E.J., Franke, C., Priller, J., Kilimann, I., Teipel, S., Buerger, K., Janowitz, D., Laske, C., Buchmann, M., Peters, O., Menne, F., Fuentes Casan, M., Wiltfang, J., Bartels, C., Düzel, E., Metzger, C., Glanz, W., Thelen, M., Spottke, A., Ramirez, A., Kofler, B., Fließbach, K., Schneider, A., Heneka, M.T., Brosseron, F., Meiberth, D., Jessen, F., and Wagner, M. (2020). Minor neuropsychological deficits in patients with subjective cognitive decline. *Neurology* 95**,** e1134-e1143.

Zhang, Z. (2017). Propensity score method: a non-parametric technique to reduce model dependence. *Ann Transl Med* 5**,** 7.
